# Supplementary material for: Supercritical Fluid Extraction of Oils from Cactus Opuntia ficus-indica L. and Opuntia dillenii Seeds
Source: Foods. 2023 Feb 1;12(3):618. doi: 10.3390/foods12030618 (PMC9914625; doi:10.3390/foods12030618)
Supplement: Supplementary file 1 [file foods-12-00618-s001.zip › foods-2173999-supplementary.pdf]

Supplementary Material

Supercritical Fluid Extraction of Oils from *Cactus Opuntia ficus-indica* L and *Opuntia dillenii* Seeds

Ghanya Al-Naqeb [1,2,*], Cinzia Cafarella [3], Eugenio Aprea [1], Giovanna Ferrentino [4,*], Alessandra Gasparini [4], Chiara Buzzanca [3], Giuseppe Micalizzi [3], Paola Dugo [3,5], Luigi Mondello [3,5,6] and Francesca Rigano [3]

1 Center Agriculture Food Environment (C3A), University of Trento, 38098 Trento, Italy
2 Department of Food Sciences and Nutrition, Faculty of Agriculture Food and Environment, University of Sana'a, Sana'a PO Box 1247, Yemen
3 Department of Chemical, Biological, Pharmaceutical and Environmental Sciences, University of Messina, 98168 Messina, Italy
4 Faculty of Science and Technology, Free University of Bozen-Bolzano, Piazza Università 5, 39100 Bolzano, Italy
5 Chromaleont s.r.l., c/o, Department of Chemical, Biological, Pharmaceutical and Environmental Sciences, University of Messina, 98168 Messina, Italy
6 Unit of Food Science and Nutrition, Department of Medicine, University Campus Bio-Medico of Rome, 00128 Rome, Italy
* Correspondence: ghanya.alnaqeb@unitn.it (G.A.-N.); giovanna.ferrentino@unibz.it (G.F.)

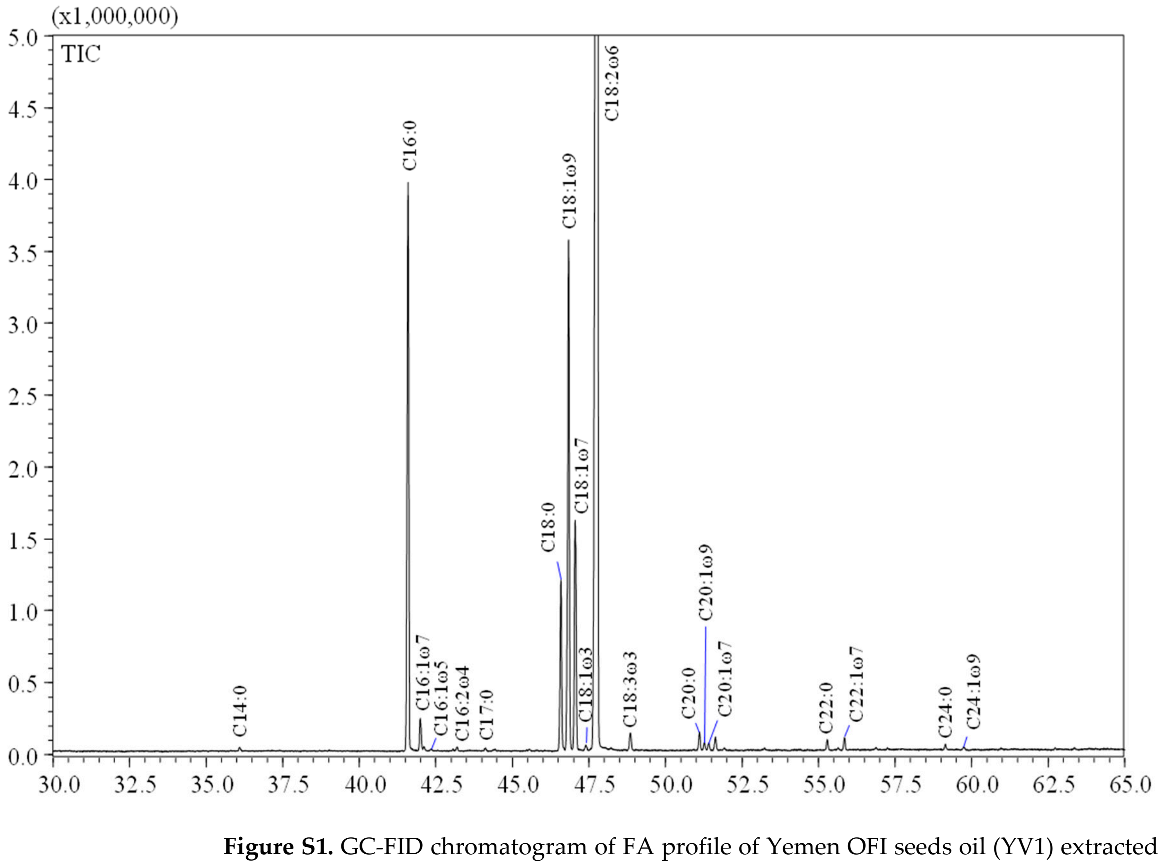

Figure S1. GC-FID chromatogram of FA profile of Yemen OFI seeds oil (YV1) extracted using SFE at 40 °C.

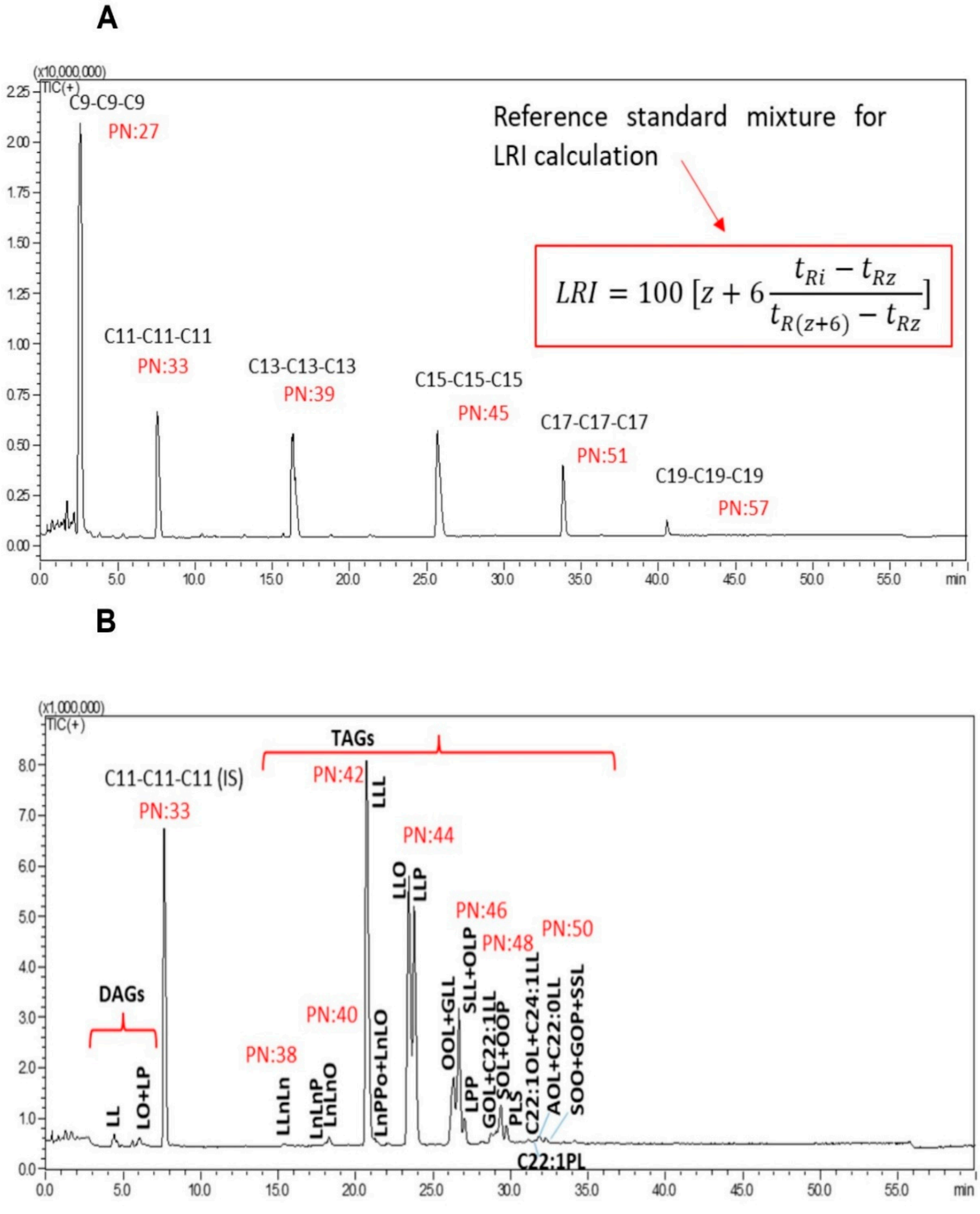

Figure S2. HPLC-MS chromatogram (**A**) and reference standard mixture (**B**) of YV1 sample.

Table S1. List of identified phenols in YV1, YV2 and I/S samples extracted by SFE and Soxhlet.

Peak *n*	Compound name	YV1 (area %)			YV2 (area %)			I/S (area %)		
		SFE 60 °C	SFE 40 °C	Soxhlet	SFE 60 °C	SFE 40 °C	Soxhlet	SFE 60 °C	SFE 40 °C	Soxhlet
1	Hydroxy-tyrosol	2.48	2.12	-	7.52	2.38	6.48	0.42	0.28	6.38
2	Tyrosol	-	2.66	-	2.65	-	1.12	1.30	2.03	0.39
3	Vanillin isomer	-	-	10.21	-	-	9.63	-	-	10.62
4	Homovanillic acid	1.28	1.31	2.33	1.38	1.20	1.02	1.07	1.72	1.32
5	Vanillic acid	17.78	13.58	17.40	16.66	20.00	11.26	18.48	18.72	12.80
6	4-hydroxybenzalde-hyde	6.65	12.71	12.04	15.30	12.04	29.17	8.71	9.51	26.59
7	Syringic acid	-	0.47	2.81	0.53	0.38	2.63	0.26	0.24	2.66
8	Vanillin	44.20	39.16	31.86	30.72	36.58	26.40	48.11	38.75	24.96
9	4-hydroxycoumarin	9.54	4.82	1.93	6.45	7.57	1.20	4.78	11.52	0.95
10	Syring-aldehyde	4.59	5.93	4.52	8.06	8.32	3.34	6.02	5.97	3.82
11	Ferulic acid	0.57	0.56	4.02	0.62	0.91	1.86	0.60	0.61	2.09
12	Cinnamic acid	0.78	0.64	1.44	0.63	0.69	0.90	0.73	0.70	0.97
13	Ferul-aldehyde	10.09	7.00	8.23	7.56	8.42	3.95	8.46	8.41	5.04
14	Sinapaldehyde	2.12	1.59	3.21	1.91	1.52	1.04	1.05	1.54	1.40
